# Middle Miocene lotus (Nelumbonaceae, *Nelumbo*) from the Qaidam Basin, Northern Tibet Plateau

**DOI:** 10.3390/biology11091261

**Published:** 2022-08-24

**Authors:** Mingyue Luo, Hui Jia, Qijia Li, Xiangning Meng, David K. Ferguson, Ping Liu, Zhuochen Han, Junjie Wang, Cheng Quan

**Affiliations:** 1 School of Earth Sciences and Engineering, Xi’an Shiyou University, Xi’an 710065, China; 2 Shaanxi Key Lab of Petroleum Accumulation Geology, Xi’an Shiyou University, Xi’an 710065, China; 3 State Key Laboratory of Palaeobiology and Stratigraphy, Nanjing Institute of Geology and Paleontology, Nanjing 210008, China; 4 School of Earth Sciences and Resources, Chang’an University, Xi’an 710054, China; 5 Department of Paleontology, University of Vienna, A-1090 Vienna, Austria

**Keywords:** *Nelumbo*, Middle Miocene, paleoecology, paleoclimate, northeast Tibetan Plateau

## Abstract

**Simple Summary:**

The new species of *Nelumbo* reported in this paper is the first fossil record of lotus in Northwest China thus far, which can provide valuable information regarding the late Middle Miocene biocoenosis composition and the paleoenvironment of the Qaidam Basin on the northern Tibetan Plateau.

**Abstract:**

The Neogene environment and paleovegetation of today’s semi-arid and arid Central Asia remain elusive. Little is known about the effect of paleoclimatic change on the distribution and ecological response mechanisms of aquatic plants, especially on the Tibetan Plateau. Here, we report a new species of *Nelumbo* Adanson, including leaves, receptacles, and fruits, namely *Nelumbo* *delinghaensis* sp. nov., from the Upper Youshashan Formation of the upper Middle Miocene in the northern Qaidam Basin on the Tibetan Plateau. The new species comprises centrally peltate leaves with 12–15 actinodromous primary veins and a receptacle embedded with ca. 15–30 fruits, with an unlobed central disc. Megafossils of lotus from northwest China broaden the geographical and stratigraphic ranges of *Nelumbo*. Our findings suggest that a large freshwater lake body surrounded by temperate forests and grassland developed in the Qaidam Basin during the late Middle Miocene, in sharp contrast to the present desert vegetation. The climate used to be sufficiently warm and moist enough to support a forest-steppe ecosystem with abundant freshwater bodies.

## 1. Introduction

Step-wise drying in Central Asia beginning in the late Eocene has been evidenced by extensive sedimentary records [1,2]. That aridity persists today, but its process and underlying mechanisms have always been controversial. Initially, a magnetic study of the Luochuan section on the Loess Plateau suggested a beginning at about 2.4 Ma [3]. In recent years, some researchers deduced that aridity occurred in the early Miocene [4,5,6,7] or formed in the late Oligocene [8,9,10,11], or even the Eocene [12,13]. The controversy of the underlying mechanisms mainly regards the roles of pCO_2_ concentrations [14], global cooling [1,15,16,17], the uplift of the Tibetan Plateau and margins associated with the India-Asia plate collision [18,19,20,21,22,23], the retreat of the proto-Paratethys [13,24,25,26], and combined effects on driving aridification.

The Qaidam Basin, the largest sedimentary basin on the northern Tibetan Plateau, has the most continuous sedimentary records in the Cenozoic [27] and provides exceptional insight into the history and inter-linkages between Central Asia aridification and regional tectonism related to the topographic development of the Tibetan Plateau. Existing evidence of climatic ecological evolution in the Cenozoic Qaidam Basin, particularly in the Neogene, is principally based on sedimentary [28,29,30,31,32], paleontological [10,30,33,34,35,36,37,38], or stable isotope [39,40,41,42] records [41,43]. For example, based on the physiognomy of plant fossils, Song et al. [44] estimate a wet temperate with a low precipitation seasonality of Qaidam in the Early Oligocene; based on sedimentological studies, Bao et al. [45] suggest a rapidly intensified aridity which is also indicated by mammal data [30].

Recently, during a geological survey in the Huaitoutala region in the northeastern Qaidam Basin, numerous plant megafossils, including leaves, fruits and seeds, were collected from the Miocene Upper Youshashan Formation (Figure 1). Aquatic plants are the dominant group among the assemblage, unlike the other fossil flora on the plateau [30,44]. Fragmentary fossil *Nelumbo* Adanson materials are preserved as leaves, fruits, and receptacles.

*Nelumbo* Adanson has an evolutionary history of ca. 135 million years and is a monotypic genus of the Nelumbonaceae, with a critical phylogenetic position in flowering plants [46]. It consists of two extremely similar extant species: *Nelumbo nucifera* Gaertn. and *Nelumbo lutea* Willd. *N. nucifera* is distributed in East, South and Southeast Asia and North Australia [47], with pink or white tepals, whereas *N. lutea* is found in Central and North America with pale yellow tepals [36]. In addition, the central disc of *N. nucifera* leaves is shallowly lobed while that of *N. lutea* leaves is deeply lobed, an important distinguishing feature between the two species. The presence of stomata on the lower surface of the leaf, seedling morphology (possessing erect stem and undeveloped taproot) [48,49,50] and plant flowering, with aerial pollination and fertilization, demonstrate that *Nelumbo* probably originated from terrestrial plants [51,52,53,54].

In this study, we focus on lotus remains collected from the Middle Miocene Huaitoutala flora and their associated sedimentary environment and discuss the late Middle Miocene paleoenvironment of the Qaidam Basin on the northern Tibetan Plateau.

## 2. Geological Background

The Qaidam Basin is the lowest but largest internally drained basin on the northeastern edge of the Tibetan Plateau, with an area of 12,000 km^2^. It is bounded by the Altyn Tagh Mountain in the northwest, the Qilian Mountains in the northeast, and the East Kunlun Mountain to the south [55] (Figure 1). The current elevation of the basin is ca. 2800–3200 m above sea level. Divided into an arid desert basin area and the surrounding alpine mountainous areas, the whole area is dominated by the plateau’s continental climate [56].

The Qaidam Basin is infilled with Cenozoic deposits with a thickness of over 15,000 m from at least the late Eocene to the present [57,58]. Based on a comparative paleomagnetic restudy and complemented by fossil vertebrates assemblage succession, Wang et al. [30] subdivided the Cenozoic strata within the Qaidam Basin into six lithostratigraphic units, in upwards order: the Lulehe Formation, Lower Ganchaigou Formation, Upper Ganchaigou Formation, Lower Youshashan Formation, Upper Youshashan Formation, Shizigou Formation, and the Qigequan Formation. The fossil material studied here occurred at the Huaitoutala town of Delingha city in Qinghai Province, China (37°14′32″ N, 96°44′9″ E, Figure 1), buried in the mudstone of the lower to the middle part of the Upper Youshashan Formation (Figure 1A). A high-resolution magnetostratigraphic study [59,60] constrains the age of the fossiliferous layer as ~12.7 Ma in the late Middle Miocene (Figure 1B).

## 3. Materials and Methods

A total of 554 specimens were collected from the fossil site. Under the prefix DLH0001-0554 for specimen numbers, all specimens are stored at the School of Earth Science and Resources, Chang’an University, Xi’an, China. Approximately 200 *Nelumbo* specimens were collected, including leaves, receptacles, and fruits preserved as coalified compression and impression remains. After preparation, all fossils were examined and photographed using a digital single-lens reflex camera (Nikon D90) and an Olympus SZ61 stereomicroscope and edited with the help of Adobe Photoshop CC. To achieve reliable identification of the fossil specimens, various extant species were critically examined using digital herbarium catalogs, viz., Kew herbarium catalog (https://apps.kw.org/herbcat/gotoCiteUs.do) (accessed on 2 March 2022). Morphological descriptions of fossil leaf specimens follow the terminology and nomenclature proposed by Ellis et al. [61].

## 4. Results

Order: Proteales Juss. ex Bercht. & J. Presl.1827.

Family: Nelumbonaceae A. Rich.1827.

Genus: *Nelumbo* Adanson. 1763.

Species: *Nelumbo delinghaensis* M. Y. Luo et H. Jia sp. nov.

Holotype: DLH550 (Figure 2A,B, Figure 3A–C and Figure 4A–D).

Paratypes: DLH156A, DLH156B, DLH551, DLH552, DLH553, DLH554, DLH560 (Figure 2C–E).

Type locality: Huaitoutala Town, Delingha City, Qinghai, China.

Stratigraphy and age: Upper member of the Upper Youshashan Formation, Middle Miocene.

Etymology: From the Delingha City where the specimens were collected.

Diagnosis: Simple, leaf centrally peltate, lamina orbiculate, margin entire or slightly sinuous, centrally positioned petiole. Primary venation actinodromous, bifurcating at least once and forming festooned brochidodromous arches; interior secondary veins intercalated with the primary veins; tertiary veins emerging from the primary veins, opposite percurrent, with sinus and straight course; quaternary venation reticulate; areolation is predominantly six-sided; marginal ultimate venation looped. Receptacles are obconical, with globose or elongated ovoid fruits.

Description:

Leaves: Simple, leaf centrally peltate, the lamina which is not fully preserved is rounded to orbicular, at least 16 to 60 cm in diameter (Figure 2A and Figure 3E,F), and often falling into the mesophyll- to macrophyll-size classes, margin entire, notched (Figure 2A). Nearly symmetric, apex rounded, circular base angled; petiole surface spinose, approximately 13–14 mm in diameter (Figure 2C). Leaves show a funnel-shaped pattern at the junction with the petiole, which is inserted centrally at the lamina, and the 12–15 primary veins emerge radially from its point of insertion (Figure 2A). Each primary vein is actinodromous at acute angles to other primary veins. The course is straight to slightly sinuous, and the veins bifurcate once at an angle of 50–70°, maintaining this course (Figure 2A,B,D). They then abruptly curve to join another primary vein or a bifurcation of a primary vein at an angle of 60–90°, forming brochidodromous arches (Figure 2A,B,D). Some veins of smaller caliber, intercalated with primary veins, are interpreted as poorly developed interior secondaries (Figure 2A and Figure 3A). These veins have deflected attachment with primary veins and intersect with tertiary veins. Tertiary veins emerge uniformly from the primary veins at right to obtuse angles consistently and is mixed percurrent, with sinus and straight course (Figure 2A,B,D,E). Quaternary venation is regular and reticulate. Apart from brochidodromous arches formed by the primary veins, there are other festooned arches formed by veins of lesser caliber, producing a looped ultimate marginal venation (Figure 2A,B,D). The areoles are well developed, mostly equiaxial, 150–550 μm in diameter and are commonly six-sided but occasionally four-, five- or seven-sided. Within the areoles, freely ending veinlets are absent (Figure 2B,D).

Receptacles and fruits: The remains of reproductive organs consist of many impressions that look inversely conical, which is an accrescent receptacle with fruits inside [62] (Figure 4). The receptacles are 2–2.5 cm long and 1.5–2.2 cm in diameter. From the distal flat portion of the receptacle arise 15–30 protuberances interpreted as fruits. Some occur individually (Figure 4B), but most are encircled by the thin wall of the receptacle, distributed densely and embedded into a single cavity which represents the remaining of the floral stigma, 2.8–3.3 mm long and 2.2–3 mm wide (Figure 4C,D). Some fruits exhibit globose or elongate ovoid bodies, while others are rounded to oblong in planiform shape, probably on account of the stronger compaction. At the tips of some fruits are a small capitate persistent stigma and a small ovate protuberance named respiratory pore (Figure 4C,D).

## 5. Discussion

The main characteristic features of the species *Nelumbo delinghaensis*, such as centrally peltate leaves, actinodromous venation and primary vein bifurcation, mixed percurrent tertiary venation, commonly six-sided areoles, and flat receptacles with fruits with a respiratory pore fruit, are known in the monotypic family Nelumbonaceae. Therefore, we can assign these leaves to the genus *Nelumbo* unambiguously. Many fossil leaves, fruits, and pollen of *Nelumbo* have been reported from the early Cretaceous and younger deposits. Their evolution and phytogeography have been extensively discussed [63,64,65,66].

**Figure 5:**
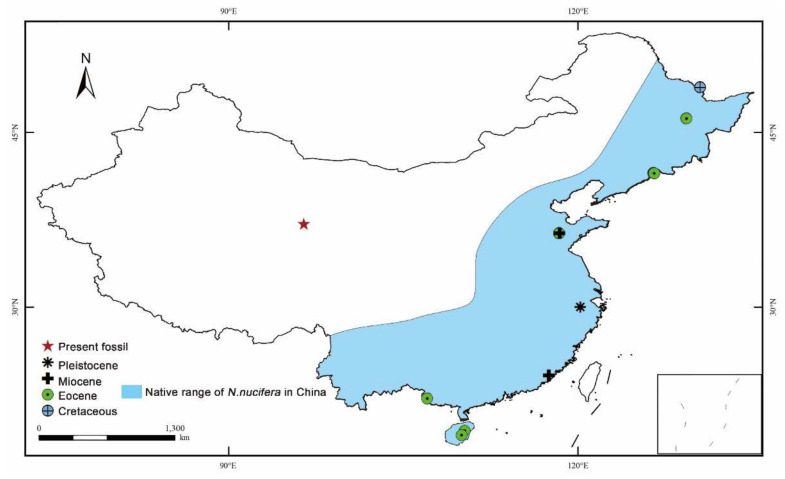
Location of the reported occurrences of *Nelumbo* in China and the present distribution of *Nelumbo* in China (modified from Borsch and Barthlott, 1994 [81]).

**Figure 1:**
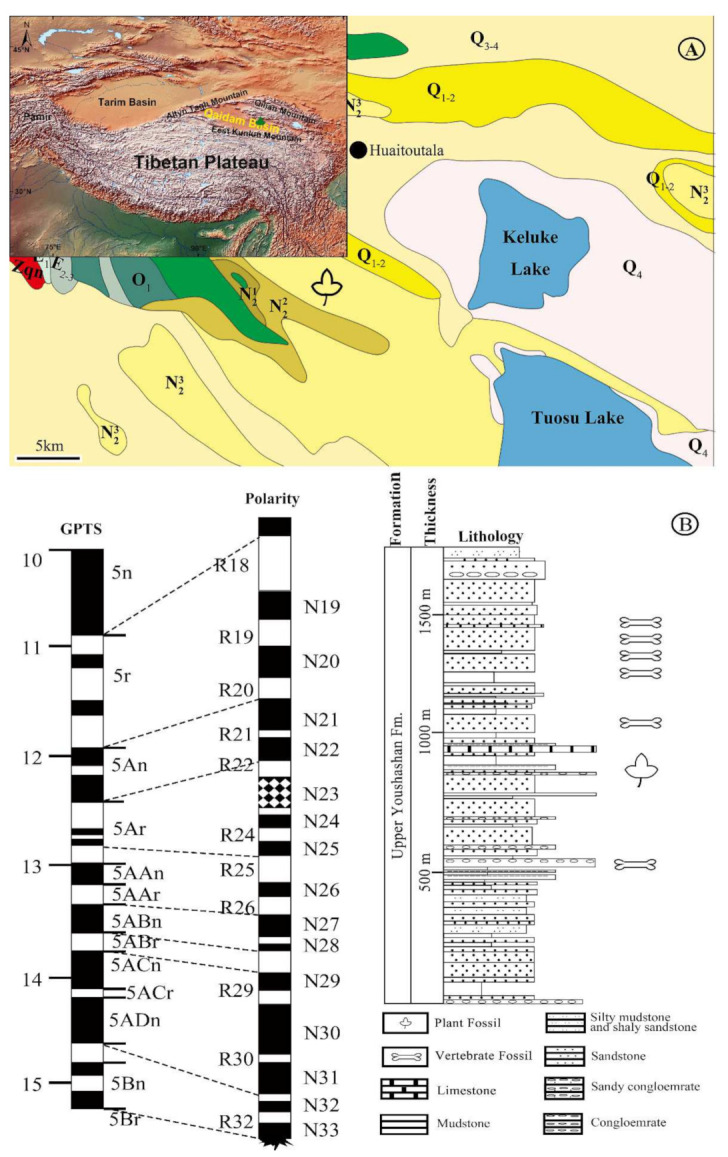
Geological setting of the fossil locality (modified from Li et al. [38]). (**A**) Maps showing the fossil location. (**B**) Stratigraphic column of the studied section. This is located in the lower to the middle part of Upper Youshashan Fm. Shown are the magnetostratigraphic correlation, the chronology and the lithology of the studied section, as well as the chronologic and stratigraphic position of the plant fossil locality.

**Figure 2:**
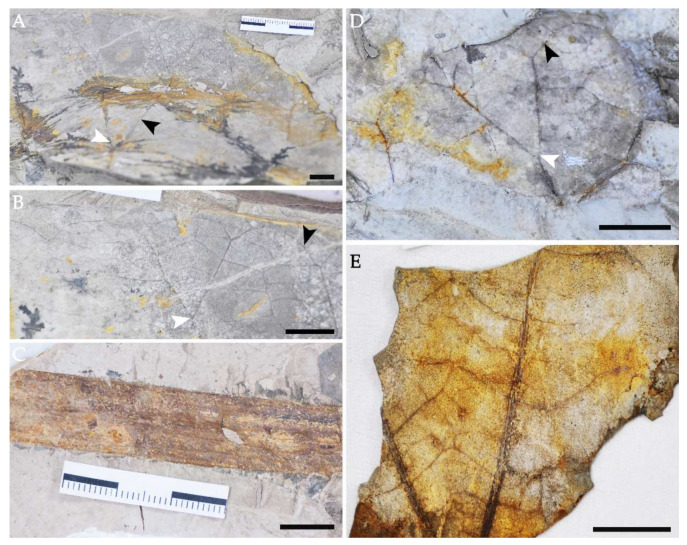
Leaves and fruit of *Nelumbo delinghaensis* from the Qaidam Basin on the Tibetan Plateau. (**A**) The general aspect of a leaf shows an orbicular and peltate lamina, radiating ribs (black arrow) from the center of leaves and the point of insertion of the petiole (white arrow). DLH550. (**B**) The counterpart leaf of (**A**) shows the position of bifurcation (white arrow) and the brochidodromous arches (black arrow). (**C**) Petiole of *N. delinghaensis* and its scattered small prickles, scale bar = 10 mm. (**D**) Another leaf shows the position of bifurcation (white arrow) and the brochidodromous arches (black arrow). DLH554. (**E**) Another leaf shows the mixed tertiary venation. DLH 552. scale bar = 10 mm.

**Figure 3:**
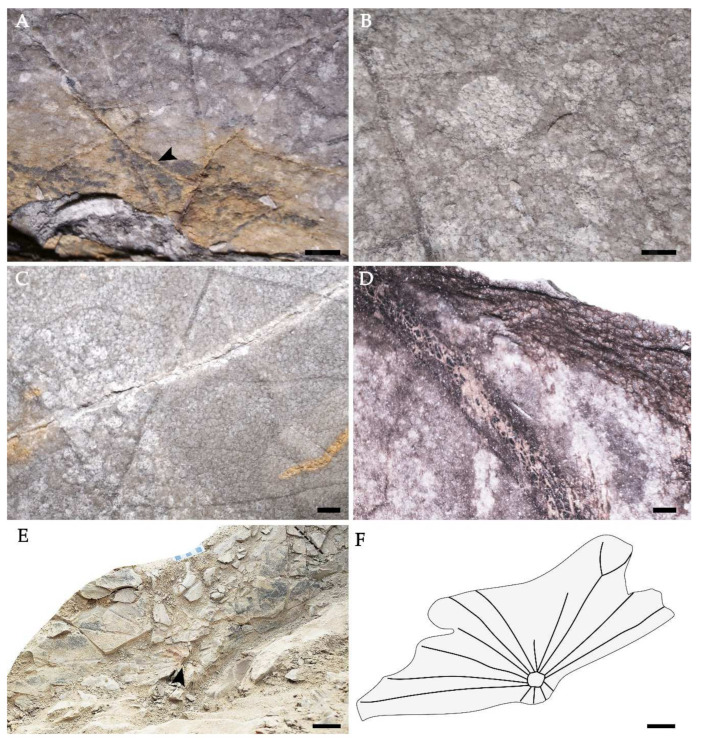
Enlarged part of the leaves of Figure 1 and the leaf of *Nelumbo delinghaensis* in situ and its line drawing. (**A**) The position of the bifurcation and poorly-developed secondary veins (black arrow). DLH550, scale bar = 5 mm. (**B**,**C**) Enlarged part of Figure 2A showing small areoles. DLH550, scale bar = 1mm. (**D**) Enlarged part of Figure 2E, scale bar = 1 mm. (**E**) Leaf of *Nelumbo delinghaensis* in situ, noting the leaf center (black arrow) and incomplete margin. (**F**) Interpretative outline drawing of *N. delinghaensis* in situ, scale bar = 50 mm.

**Figure 4:**
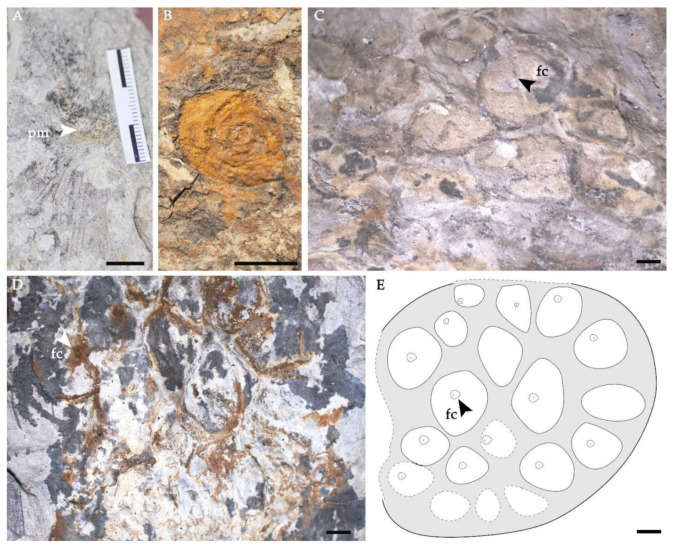
Receptacles, fruits and petiole of *Nelumbo delinghaensis* sp. nov. (**A**) Receptacle and the point of insertion of the stalk. DLH551, scale bar = 10 mm. (**B**) An individual fruit. DLH560. (**C**,**D**) Top view of receptacles with fruits. DLH156A, scale bar = 2 mm. (**E**) Interpretative outline drawing of receptacle and fruits, scale bar = 2 mm. (pm: peduncle mould; fc: fruit cavities).

In China, lotus had a wide distribution and diverse species from the Cretaceous to the Miocene. To date, the fossil record of lotus extends from north to south, north to the Eocene Dalianhe Formation and the Upper Cretaceous Yong’ancun Formation in Heilongjiang Province [67,68], south to the Eocene Changchang Formation in Hainan Province [69,70], and east to the Miocene Fotan Group in Fujian Province [71]. However, there were a few reports in the western region of China, especially in the arid northwest. The present discovery represents the most western occurrence of this genus in China and the first fossil record of lotus in northwest China thus far (Figure 5).

### 5.1. Comparisons

Four extinct genera in Nelumbonaceae have been described to date, namely *Nelumbites* Berry [72], *Paleonelumbo* Knowlton [73], *Nelumbago* McIver et Basinger [74] and *Exnelumbites* Estrada-Ruiz, Upchurch, Wolfe and Cevallos-Ferriz [75] (Table 1). Berry [72] erected the genus *Nelumbites* for the first time on the basis of fossil leaves from the Early Cretaceous Patapsco Formation of the Potomac Group on the Atlantic Coastal Plain. *Nelumbites*, with entire to crenate or crenulate margins, eccentric peltate leaves, reticulate tertiary venation, and small size, differ from the leaves of *Nelumbo delinghaensis*. Knowlton [73] established the genus *Paleonelumbo* on the basis of the leaves from the Late Cretaceous to Paleocene Dawson Arkose of Colorado, USA. It is thought to be similar to extant *Nelumbo* and consists of one species, *P. macroloba*. *Paleonelumbo* displays a toothed or lobed margin with glands, no bifurcating primary veins, and orthogonal reticulate tertiary venation, while *N. delinghaensis* has an entire margin with bifurcating primary veins. From the early Paleocene of the Ravenscrag Formation, Canada, *Nelumbago* morphologically resembles *N. delinghaensis* and extant *Nelumbo* but differs in having reticulate rather than percurrent tertiary and higher order venation, predominantly quadrangular rather than hexagonal areolation, and it lacks a central disc [74]. Later, Estrada-Ruiz et al. [75] described *Exnelumbites* from the Late Cretaceous (Campanian-Maastrichtian) Olmos Formation of Coahuila, Mexico and Jose Creek Member of the McRae Formation of south-central New Mexico, USA. *Exnelumbites* displays features including a toothed glandular margin, absent disc, no bifurcating primary venation, alternate percurrent to reticulate tertiary venation, and polygonal areolation. As can be seen in Table 1, with the exception of the central insertion of the petiole and laminar size, all these characters are absent from *Nelumbo* and *N. delinghaensis*.

To precisely determine the characteristics of *Nelumbo*-like fossil leaves, both Upchurch et al. [63] and Estrada-Ruiz et al. [75] listed the foliar features of extant *Nelumbo*, and the latter provided a more elaborate description. Afterward, Li et al. [76] made a detailed comparison of the two extant species of *Nelumbo*, not only regarding morphology but also cuticle and epicuticular ultrastructure. Descriptions from Estrada-Ruiz et al. [75] can be briefly summarized as follows: (1) mesophyll or macrophyll size, entire margin, peltate, orbicular lamina, and centrally positioned petiole; (2) a bilaterally lobed central disc; (3) primary venation is actinodromous, with 18–24 primary veins. One vein named the midvein shows no branching, is straight and runs directly to the leaf margin, which can be used to define the line of symmetry of the lobed central disc; other primary veins dichotomize 2–3 times near the margin and form an inner and outer set of intramarginal loops; (4) tertiary veins, interconnected with primary veins, are mixed percurrent; (5) quaternary venation is mixed percurrent; (6) areolation is isodiametric and predominantly hexagonal, with a mix of six- and five-sided areoles; freely ending veinlets are absent. It should be pointed out that Upchurch et al. [76] described the leaf morphology of extant *Nelumbo* merely based on the species-*N. lutea*, but the information is still applicable because of the similarity of the macrostructure of *Nelumbo* [76]. The new fossil leaves are similar to the two living species in terms of peltate orbicular leaves, a central petiole, the number of radiating veins, dichotomous branching, and the obconical receptacles with nut-like fruits [76,77,78,79]. The number of primary veins [80] and arrangement of the fruits are considered diagnostic characteristics in fossil species of *Nelumbo*. A detailed comparison indicates (Table 2) that leaves of *N. delinghaensis* possess fewer primary veins and are smaller in size and display poorly developed secondary veins that are absent in the leaves of extant ones. Moreover, the fruits of *N. delinghaensis* are fewer in number and smaller in size in each receptacle. In addition, the leaves of *N. delinghaensis* also differ from the extant ones in the central disc, which is not lobed.

The earliest geological records described as belonging to *Nelumbo* are *N. lusitanica* Saporta, and *N. choffati* Saporta from the Albian region of Portugal, and the leaf of *N. weymouthi* Brown reported from the Aspen Shale of southwestern Wyoming, USA [64,82,83]. Since then, more than 30 fossil species have been reported, of which 18 species are known from the Cretaceous of Europe, Asia, North America, South America and Africa, with the rest recorded from the Paleogene and the Neogene of Asia, Europe, and North America [51,68]. Most species were established based on a single specimen or just a mere fragment during the 20th century [68,70,71,79], such as *N. lacunosa*, *N. megalopolitana* and *N. minima* erected for fossil receptacles [51]. Due to the lack of detailed descriptions and comparisons of some fossils, a more thorough investigation is required to clarify the taxonomic position of these fossils. Therefore, some undisputed fossil species of *Nelumbo* are compared with the present specimens, including *N. changchangensis*, *N. protospeciosa*, *N. jiayinensis*, *N. fotanensis*, *N. orienlalis* and *N. puertae*. The first four were reported from China.

*Nelumbo jiayinensis* was described from the Upper Cretaceous Yong’ancun Formation, Heilongjiang, Northeast China, the earliest known age and the most northern occurrence of this genus in China. *N. jiayinensis* differs from our specimens in the size of areoles and the mode of areole formation and lacks bifurcating primary venation [68]. *Nelumbo changchangensis* was erected by He et al. [70] with a comprehensive record from the Eocene Changchang Formation, Changchang Basin, Hainan Island, China, including rhizomes, tubers, leaves, receptacles and fruits. The species bears similarities to *N. delinghaensis* in the size and shape of areolation and laminar size, but the number of ribs, the size of fruits and receptacles, the secondary venation, and the nature of the areoles are different. *N. protospeciosa* from the Eocene Linjiang Formation of Jiangxi, China [84], was originally described from the Aquitanian region of southern France by Saporta [85]. The primary venation of *N. protospeciosa* gives rise to transverse or crooked secondary veins, as in *N. delinghaensis*; however, the angles of bifurcations are much smaller than those of the present specimens. *N. fotanensis*, described from the Miocene Fotan Formation of Fujian, China [71], is characterized by having inner and outer loops near the margin formed by primary venation, percurrent tertiary venation, no lobed central disc, and the size and shape of areolation, fruits and receptacles. However, it differs from *N. delinghaensis* in the number of primary veins, laminar size, and the angles and course of bifurcations. *N. puertae*, the oldest fossil record of *Nelumbo* in the Southern Hemisphere, was described by Gandolfo and Cúneo [79] from the Upper Cretaceous of Chubut Patagonia, Argentina. Both *N. puertae* and *N. delinghaensis* have poorly developed secondary venation, but in *N. delinghaensis*, the tertiary venation is mixed rather than opposite percurrent, and the quaternary venation is reticulate rather than opposite percurrent. Laminar size and shape of the areolation of the present specimens are similar to those of *N. orientalis* from the Upper Cretaceous of Japan [80]; however, the number of radial veins and the angles of bifurcations are larger than those in *N. orientalis*.

**Figure 6:**
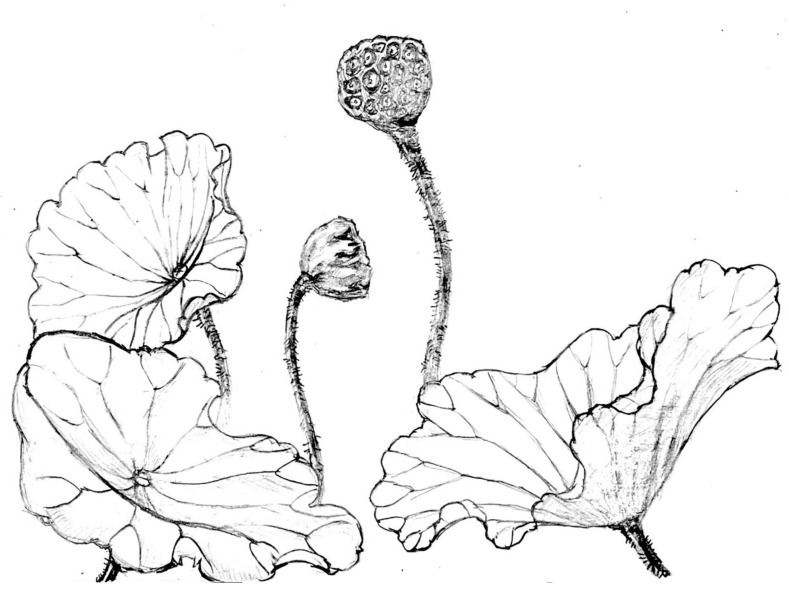
Reconstruction of *Nelumbo delinghaensis* sp. nov. (drawn by Junjie Wang, one of the authors of this article).

Based on the comparative analysis, the present fossil should be assigned as a new species of *Nelumbo* named *Nelumbo delinghaensis* M. Y. Luo et H. Jia sp. nov. with almost all the synapomorphies of extant *Nelumbo*. In the Miocene deposits where the new species was discovered, Nelumbo’s organs, except flowers and rhizomes, were collected. The combination of these organs enables a relatively whole-plant reconstruction of this new species (Figure 6).

### 5.2. Paleoenvironmental Significance

The Qaidam Basin is a key area for studying uplift and environmental change of the plateau from the continuous Cenozoic sedimentary records on the northern Tibetan Plateau. Various types of fossils have been preserved in the strata, for example mammals [30,85,86,87,88], insects [89], fish [35,90,91], ostracodes [92,93], spore pollen [16,37,94], plants [38,95,96] and even trace fossils [97,98]. Previous knowledge about vegetation and the environment in the Qaidam Basin during the Miocene has been acquired from palynological and paleontological studies [96]. Wang et al. [30] established a faunal sequence from the early Oligocene to the early Pliocene for the first time by extensively collecting paleozoological data in the central and eastern parts of the basin and deduced that the eastern Qaidam Basin in the late Middle Miocene featured a mixed habitat of open and wooded environments with abundant freshwater streams. Palynological assemblages also show forest-steppe vegetation in the Miocene [94]. However, fossil records of plants, especially aquatic plants, are scarce. Present aquatic plant fossils found in the strata of the Upper Youshashan Formation from the Huaitoutala area in the northern Qaidam Basin provide valuable materials and evidence for a comprehensive and in-depth understanding of the paleoenvironment and paleoclimate in the basin.

As mentioned above, the genus *Nelumbo* comprises two modern species: *N. nucifera* and *N. lutea*. They are now widespread in the subtropics and temperate zone of Southeast Asia, North Australia and North America. Because of their special growth habits, both *Nelumbo* species are clear indicators of non-marine freshwater aquatic environments, such as lakes, swamps, ponds, slowly flowing streams or river margins. *Nelumbo delinghaensis*, which bears the common characteristic of the two extant species (mentioned above), probably lived in a similar environment. At the same time, a large body of freshwater is also evidenced by the co-occurrence of other fossils of aquatic plants, such as *Phragmites* Adans., *Typha* Linn., and *Equisetum* Linn. *Trapa* Linn., and aquatic animals. Biomarkers, stable isotopes, and pollen concentration reports have also suggested that a relatively large lake body was present in the Qaidam Basin during the middle-late Miocene [42,99,100]. The Miocene Huaitoutala flora, with many arthropod-damaged leaf fossils, grew near the lake [38]. In addition, a forest habitat existed close to the lake, as indicated by mammals (i.e., *Lagomeryx* Rogar and *Stephanocemas* Colbert) [30] with a preference for wooded environments. Some mammals (i.e., *Acerorhinus* Kretzoi), insects (i.e., *Aedes* and *Syrphus* Matsumura et Adachi) [86,89] and leaves damaged by arthropods [38] indicate the existence of scrubland and open steppe. Based on all the evidence mentioned above, the inferred habitat reflects a flourishing ecosystem. Fish and aquatic plants such as *Nelumbo* and *Trapa* lived in a lake surrounded by a forest. Several plants with a swampy habit, for example, *Phragmites*, *Typha*, and *Equisetum*, developed in the shallow water near the lakeshore. Shrubs grew in semi-shade or moist, dappled sunlight in temperate forests. Evidence based on geochemical proxies and fossils [30,39,41,45,101] also suggests a relatively warm and humid climate during ca. 15.3–12 Ma. Therefore, the present fossils probably lived in a lacustrine environment. The lush vegetation growing in the warm and humid temperate climate is in sharp contrast to today’s arid desert environment.

Moreover, three types of fossil woods and new chalicothere fossils reported from the Upper Youshashan Formation in the Nanbaxian and Quanshuiliang areas, respectively, indicate that the temperate deciduous broad-leaved forest and grasslands mixed with woodland were still growing in the Qaidam Basin during the middle-late Miocene [34,96]. The Neogene Zekog flora also points to temperate deciduous broad-leaved forest dominating the eastern Qaidam Basin during the Miocene [102].

To summarize, the fossil lotus in the northern Qaidam Basin, together with the fossil leaf assemblages and mammals, insects, and woods found within the basin, signal that a large lake body once occurred in the Qaidam Basin and the temperate deciduous broad-leaved forests once grew in the Qaidam Basin and adjacent regions on the northern Tibetan Plateau in the Miocene, co-existing with grassland vegetation. The climate was sufficiently warm and moist enough to support a forest-steppe ecosystem with abundant freshwater bodies.

## 6. Conclusions

In this paper, we described a new species, *Nelumbo delinghaensis* M. Y. Luo et H. Jia sp. nov., from the late Middle Miocene Upper Youshashan Formation of Huaitoutala section, Qinghai, Northwest China. The fossil species comprises not only vegetative organs but also reproductive organs and possesses all the characteristics of extant *Nelumbo* in terms of leaf architecture and fruit morphology. It is morphologically similar to *N. nucifera*. The new species represents the westernmost occurrence of this genus in China and the first fossil record of lotus in Northwest China, thus extending the geographical and stratigraphic ranges of *Nelumbo*. Our results show that a freshwater lake body surrounded by temperate forests and grassland once occurred in the Qaidam Basin during the late middle Miocene, in sharp contrast to the desert vegetation that exists today.

## Figures and Tables

**Table 1:** Comparisons of leaf features between *Nelumbo* and the stem group of Nelumbonaceae.

	Character	*N. delinghaensis*	*Nelumbo* (Extant)	*Nelumbites*	*Paleonelumbo*	*Nelumbago*	*Exnelumbites*
Taxon							
Leaf margin		Entire	Entire	Entire to crenate or crenulate	Toothed or lobed with gland	Entire	Toothed with gland
Central disc		Present	Present	Absent	Absent	Absent	Absent
Position of petiole		Centrally peltate	Centrally peltate	Eccentrically peltate	Centrally peltate	Centrally peltate	Centrally peltate
Primary venation		Bifurcated, 12–15	Bifurcated, over 18	Distinct midvein No bifurcation, fewer than 10	No bifurcation, 10–15	Bifurcated, over 20	No bifurcation, 12–13
Secondary venation		Present	Absent	Present	Present	No data	Present
Tertiary venation		Irregular, mixed percurrent	Regular, opposite percurrent	Irregular, reticulate	Orthogonal reticulate	Irregular, orthogonal reticulate	Irregular, alternate percurrent to reticulate
Quaternary venation		Regular reticulate	mixed percurrent	Reticulate	Percurrent	Orthogonal reticulate	Reticulate
Areoles		Predominantly Hexagonal	Predominantly Hexagonal	Polygonal	No data	Commonly quadrilateral	Polygonal
Size		Mesophyll to macrophyll	Mesophyll to macrophyll	Notophyll to microphyll	Mesophyll	Notophyll to mesophyll	Mesophyll

**Table 2:** Morphological comparison of leaves, receptacles and fruits in *Nelumbo delinghaensis* and extant species.

Organ	Character	*N. delinghaensis*	*N. nucifera*	*N. lutea*
Leaf	Number of primary veins	12–15	20–25	20–25
	Diameter	8–30 cm	7–85 cm	60 cm
	Shape of areoles	4- to 7-sided	4- to 7-sided	4- to 7-sided
	Size of areoles	150–550 μm	129–661 μm	148–634 μm
	Central disc	Present	Present, shallowly lobed	Present, deeply lobed
	Highest order venation	4°	4°	4°
Fruit	Size	2.8–3.3 mm × 2.2–3 mm	18 × 10 mm	No data
	Number of fruits	15–30	1–40	12–25
Receptacle	Size	2–2.5 cm × 1.8–2.3 cm	3.8–4.5 cm × 7.5–9.6 cm	No data
	Shape	Obconical	Obconical	Obconical
Reference			(Li et al., 2016 [46]; Fu&Wiersema, 2001 [77])	(Li et al., 2016 [46]; Hall & Penfound, 1944 [78]; Gan-dolfo & Cúneo, 2005 [79])

## Data Availability

All data dealing with this study are reported in the paper.
